# LL-37 Might Promote Local Invasion of Melanoma by Activating Melanoma Cells and Tumor-Associated Macrophages

**DOI:** 10.3390/cancers15061678

**Published:** 2023-03-09

**Authors:** Kentaro Ohuchi, Tetsuya Ikawa, Ryo Amagai, Toshiya Takahashi, Yuna Roh, Junko Endo, Yumi Kambayashi, Yoshihide Asano, Taku Fujimura

**Affiliations:** Department of Dermatology, Tohoku University Graduate School of Medicine, Sendai 980-8574, Japan

**Keywords:** LL37, melanoma, tumor stage, angiogenesis

## Abstract

**Simple Summary:**

LL-37 contributes the vertical invasion of tumor cells in melanoma. Indeed, the ratio of LL-37-expressing cells correlated positively to T stage severity. Moreover, LL-37 induced pro-angiogenic factors in both human and mouse systems. Our results suggested that LL-37 stimulates both tumor cells and macrophages to promote melanoma invasion by the induction of pro-angiogenic factors.

**Abstract:**

LL-37 can stimulate various skin-resident cells to contribute to tumor development. Since tumor (T) stage is determined by the vertical invasion of tumor cells in melanoma, we hypothesized that the LL-37 expression level is correlated with the T stage in melanoma patients. Immunohistochemical staining of LL-37 was performed in each stage of melanoma (Tis-T4), suggesting the ratio of LL-37-expressing cells correlate positively to T stage severity. Next, to examine pro-angiogenetic factors induced by LL-37 stimulation, the B16F10 melanoma model was used. Intra-tumorally administered CRAMP, the mouse ortologe of LL-37, significantly increased the mRNA expression of *CXCL5*, *IL23A*, *MMP1a*, and *MMP9* in B16F10 melanoma. To confirm the induction of pro-angiogenic factors, A375 human melanoma cells were stimulated by LL-37 in vitro. The mRNA expression of *CXCL5*, *IL23A*, and *MMP9*, but not *MMP1*, were significantly increased by LL-37 stimulation. Moreover, LL-37-stimulated A375 culture supernatant promoted tube networks, suggesting that these tumor-derived factors promote the pro-angiogenic effect on tumor development. In contrast to melanoma cell lines, M2 macrophages stimulated by LL-37 in vitro significantly increased their expression and secretion of MMP-1, but not MMP-9 expression. Collectively, these results suggest that LL-37 stimulates both tumor cells and macrophages to promote melanoma invasion by the induction of pro-angiogenic factors.

## 1. Introduction

LL-37 is an autoantigen derived from keratinocytes that can bind to surface scavenger receptors expressed on myeloid dendritic cells (DCs), plasmacytoid DCs, and macrophages to recognize extracellular self-DNA [1,2,3]. Among such scavenger receptors, CD163 is a specific surface marker of macrophages [4,5]. The expression of LL-37 on myeloid cells in dermis is correlated with levels of proinflammatory cytokines such as IL-23p19, IL-17A, and TNF-α [6,7], which could promote tumor progression via angiogenesis in melanoma and non-melanoma skin cancers [8,9,10,11]. Moreover, LL-37 is expressed in melanoma cells, potentially promoting their own proliferation, migration and invasion by autocrine and/or paracrine fashions [11,12,13]. Notably, dermal expression of LL-37 is increased in parallel with dermal invasion of tumor cells in skin cancers [14,15].

LL-37 is classically known as an antimicrobial peptide that possesses immunomodulatory and stimulatory effects on various skin-resident cells such as neutrophils, T cells and macrophages [16,17,18]. LL-37 stimulates these skin-resident cells to produce various chemokines, leading to recruit further immune cells to develop inflammatory microenvironment in the lesional skin of cutaneous disorders [18,19]. Notably, genetically lack of CRAMP (the mouse ortologe of LL-37) significantly reduces the neutrophils recruitment in muscle in the mouse Duchenne muscular dystrophy model [20], though its effects on skin inflammatory disorder is still controversial [21]. In addition to immunomodulatory effects, LL-37 promotes angiogenesis in the skin by the induction of vascular endothelial growth factor (VEGF) and matrix metalloproteinases (MMPs) [19]. Importantly, these angiogenetic factors from tumor cells as well as tumor associated macrophages (TAMs) play significant roles in development of skin cancers. Indeed, dermal expression of LL-37 as well as MMPs are increased in parallel with the dermal invasion of tumor cells in extramammary Paget’s cells in the skin and cutaneous squamous cell carcinoma [14,15]. Collectively, since LL-37 might be correlated with tumor progression in skin cancers, in this report, the expression of LL-37 in melanoma was investigated, along with possible local invasion mechanisms of melanoma through pro-angiogenesis via LL-37 pathways.

## 2. Materials and Methods

### 2.1. Reagents

Synthetic LL-37 peptides were synthesized and purchased from Genemed Synthesis Inc. (San Antonio, TX, USA). For immunohistochemical staining, monoclonal Abs against human LL37 (Santa Cruz, Dallas, TX, USA) and mouse anti-human CD163 phycoerythrin-conjugated monoclonal antibody (R&D Systems, Minneapolis, MN, USA) were used.

Tissue sample collection, immunohistochemical staining, and immunofluorescence staining were also carried out.

Archived formalin-fixed, paraffin-embedded skin specimens from melanoma patients treated in the Department of Dermatology at Tohoku University Graduate School of Medicine, Sendai, Japan, were collected. All patients gave their informed consent. The study was approved by the Ethics Committee of Tohoku University Graduate School of Medicine (permit number: 2021-1-1213) and conducted according to the guidelines of the Declaration of Helsinki.

The 10 non-invasive melanomas in each stage were processed for single staining of LL-37 (Table 1). Briefly, formalin-fixed, paraffin-embedded tissue samples were sectioned at 4 µm and deparaffinized. Antibody binding was demonstrated via alkaline phosphatase-conjugated anti-mouse Ig (Histofine SAB-AP(R) kit; Nichirei, Tokyo, Japan) for anti-LL37 Abs or their isotype controls. To quantify the immunohistochemical staining of each sample, positive cells were counted using a BZ-X800 microscope (KEYENCE, Tokyo, Japan). The percentage of IHC-positive cells per all tumour-infiltrating cells was counted automatically [14].

For cryosections, each sample was frozen in optimal cutting temperature embedding medium, and 6-µm-thick sections were fixed with cold acetone for 30 min and then blocked with IF buffer (PBS, 5% bovine serum albumin). Thereafter, each section was incubated with the relevant antibodies. The slides were mounted in DAPI Fluoromount g (Southern Biotech, Birmingham, AL, USA) and examined using a BZ-X800 microscope.

### 2.2. Tumor Inoculation and Treatment

B16F10 melanoma cells (100 μL of 2 × 10^6^ cells/mL) were subcutaneously injected into female C57BL/6 mice, as described previously [22]. For qRT-PCR analysis, CRAMP (mouse LL-37) (20 μg/mouse) was peritumorally injected on day 7, and the tumor was harvested on day 9. For qRT-PCR, whole tumor was frozen with liquid nitrogen and then crushed with Cryo-Press (MICROTEC, Chiba, Japan). Total RNA was extracted using ISOGEN (NIPPON GENE, Tokyo, Japan) according to the manufacturer’s instructions. The protocol for the animal study was approved by the ethics committee at Tohoku University Graduate School of Medicine for Animal Experimentation, Sendai, Japan (permit number: 2019MdLMO-134-03). The research complied with the Tohoku University Graduate School of Medicine’s Animal Experimentation Ethics guidelines and policies.

### 2.3. Cell Lines and Cell Culture

Human melanoma, A375, cell lines were purchased from the American Type Culture Collection (Manassas, VA, USA). The culture medium contained DMEM (Sigma-Aldrich, St. Louis, MO, USA) supplemented with 10% fetal bovine serum (Biological Industries, Kibbutz Beit Haemek, Israel), penicillin (100 units/mL), streptomycin (0.1 mg/mL), and amphotericin B (0.25 μg/mL). A375 cells were stimulated without or with 0.2 mM or 1 mM LL37. Cells were harvested 6 h after stimulation for RNA extraction. The supernatants were collected 48 h after stimulation for Western blot, ELISA assay, and tube formation assay.

### 2.4. RNA Extraction and Quantitative Real-Time PCR Experiments

Total RNA was extracted using an RNeasy Micro kit (Qiagen, Courtaboeuf, France) in accordance with the manufacturer’s instructions. RNA was eluted with 14 μL of RNase-free water. DNase I treatment (RNase-Free DNase Set; Qiagen) was performed to remove contaminating genomic DNA. Reverse transcription was performed with the SuperScript VILO cDNA Synthesis kit (Invitrogen, Carlsbad, CA, USA). Amplification reactions were performed using an Mx 3000P Real-Time Quantitative PCR System (Stratagene, San Diego, CA, USA). Relative mRNA expression levels were calculated for each gene and each time point after normalization against GAPDH using the ΔCt method or ΔΔCt method.

### 2.5. Western Blotting

A375 cells were seeded onto 6-well plates and cultured as described above. Cells were collected and disrupted in lysis buffer (Cell Signaling Technology, Boston, MA, USA). After adding SDS sample buffer (Cell Signaling Technology), lysates were electrophoretically separated on a 12% polyacrylamide gel (ATTO Corp., Tokyo, Japan). Proteins were electrophoretically transferred onto a polyvinylidene difluoride membrane (Bio-Rad, Hercules, CA, USA). The membrane was blocked in 5% nonfat dry milk in Tris-buffered saline (TBS) with 0.1% Tween-20 (TBST) for 1 h at room temperature. After several washes with TBST, the membrane was incubated overnight at 4 °C with primary mouse anti-human IL-23p19 antibody (Lifespan Bioscience, 1:1000) or mouse anti-human beta-actin antibody (Cell Signaling Technology, Tokyo, Japan; 1:1000). The membrane was washed several times in TBST followed by 1 h incubation with horseradish peroxidase–conjugated goat anti-mouse IgG secondary antibody (Santa Cruz, CA, USA).

### 2.6. In Vitro Angiogenesis Assay

Human dermal microvascular endothelial cells (HDMECs) (Lonza, Zurich, Switzerland) were co-cultured with culture supernatant of A375 treated with or without LL-37 (1 mM) alone for 48 h. Then, cells were treated with 10 μg/mL of mitomycin C (Sigma Aldrich, Tokyo, Japan) for 2 h. A 24-well plate was coated with 250 μL of growth factor-reduced Matrigel (BD Biosciences, San Jose, CA, USA). After the gel was solidified, cells were trypsinized and seeded onto the Matrigel at 7 Å, approximately 10^4^ cells per well, and incubated for 24 h. Cells were treated with calcein AM before observation [23]. Six photographs were taken randomly from each well. The area of meshes and tube formation was calculated using a BZ-X800 microscope (KEYENCE) [14].

### 2.7. Culture of M2 Macrophages from Human Peripheral Blood Monocytes

CD14^+^ monocytes were isolated from peripheral blood mononuclear cells from healthy donors using MACS beads (CD14 microbeads, Miltenyi Biotec Inc., Sunnyvale, CA, USA) according to the manufacturer’s protocol. CD14^+^ monocytes (2 × 10^5^/mL) were cultured in complete medium containing 100 ng/mL of recombinant human M-CSF for 5 days, as previously reported [24,25]. On the fifth day, monocyte-derived macrophages were treated with or without graded LL-37 for 48 h, and culture supernatant was harvested.

### 2.8. Cytokine Enzyme-Linked Immunosorbent Assays (ELISAs)

The culture supernatants were collected as described in materials and methods, and the levels of secreted CXCL5, MMP-1, MMP-9, and IL-23p19 proteins were determined by ELISA, according to the manufacturer’s protocol (R&D Systems).

### 2.9. Statistical Analysis

For a single comparison between two groups, the Mann–Whitney U-test was used. For multi-group comparisons, the Kruskal–Wallis test was used. The level of significance was set at *p* < 0.05.

## 3. Results

LL-37 expression was increased in parallel with T stage in melanomas.

**Figure 1:**
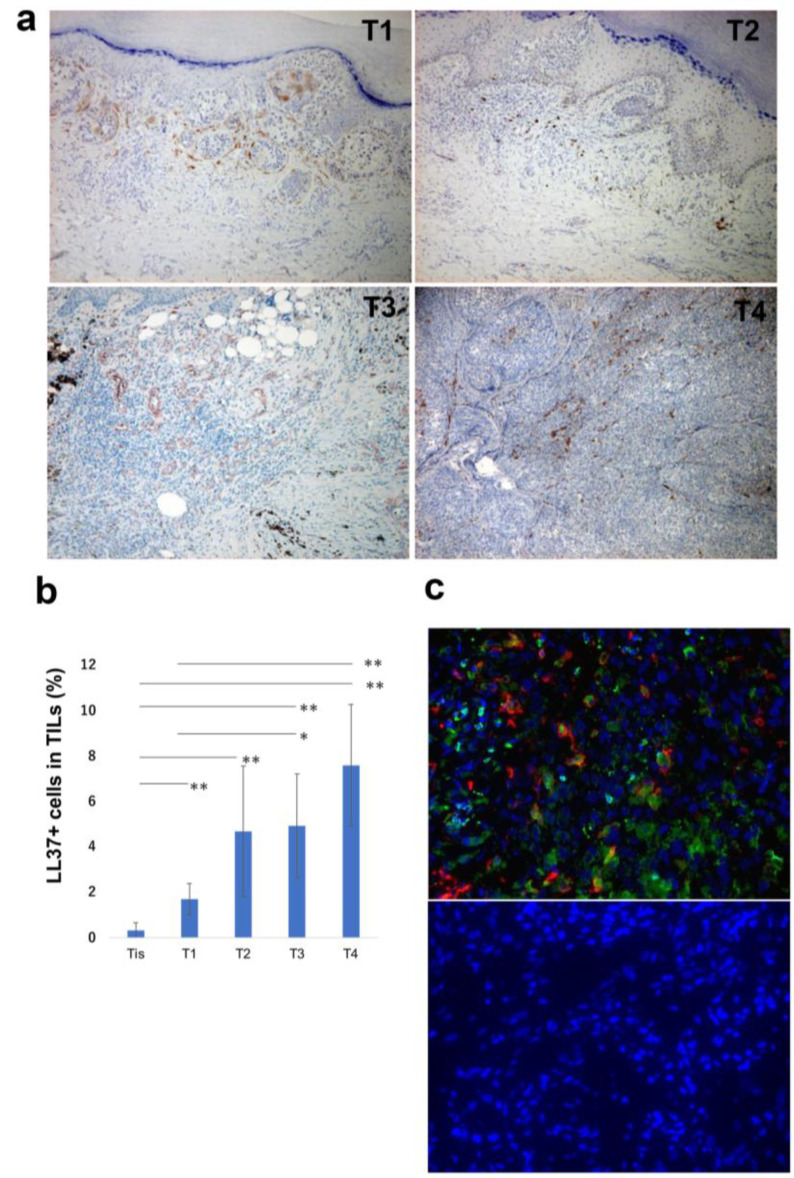
Representative paraffin-embedded tissue samples from lesional skin of patients with melanoma in each tumor stage (**a**). Quantitative analysis of LL-37+ cells: the IHC-positive cells within the lymphocyte fraction and the percentage of IHC-positive cells per all tumour-infiltrating cells were automatically counted using a BZ-X800 microscope (**b**). IF staining of melanoma for LL-37 (green), CD163 (red), and DAPI (blue, nuclei). A merged image is also shown, with green and red combining into yellow. The isotype control IgG1 stains as red or green (**c**). * marks a significant (*p* < 0.05) difference. ** marks a significant (*p* < 0.01) difference.

As we previously reported, since the expression of LL-37 was increased in parallel with the dermal invasion of various skin cancers such as cutaneous squamous cell carcinoma [14], we hypothesized that levels of LL-37 correlate with the dermal invasion of melanoma cells. To test this, immunohistochemical staining of 10 invasive melanomas in each T stage (T1-T4) (Figure 1a) and 10 melanoma-in situ patients was performed (Table 1). Numbers of LL-37+ cells were counted by a BZ-X800 digital microscope as we previously reported [14]. The ratio of LL-37+ cells in tumor-infiltrating leukocytes (TILs) was significantly increased in parallel with tumor stage (Figure 1b). Immunohistochemical staining showed that LL-37 was expressed on CD163+ macrophages (Figure 1c), in addition to melanoma cells, as previous reports suggested [11]. 

Intra-tumor injection of LL-37 significantly increased *CXCL5*, *IL23A*, *MMP1a*, and *MMP9* mRNA expression in mouse B16F10 melanoma.

**Figure 2:**
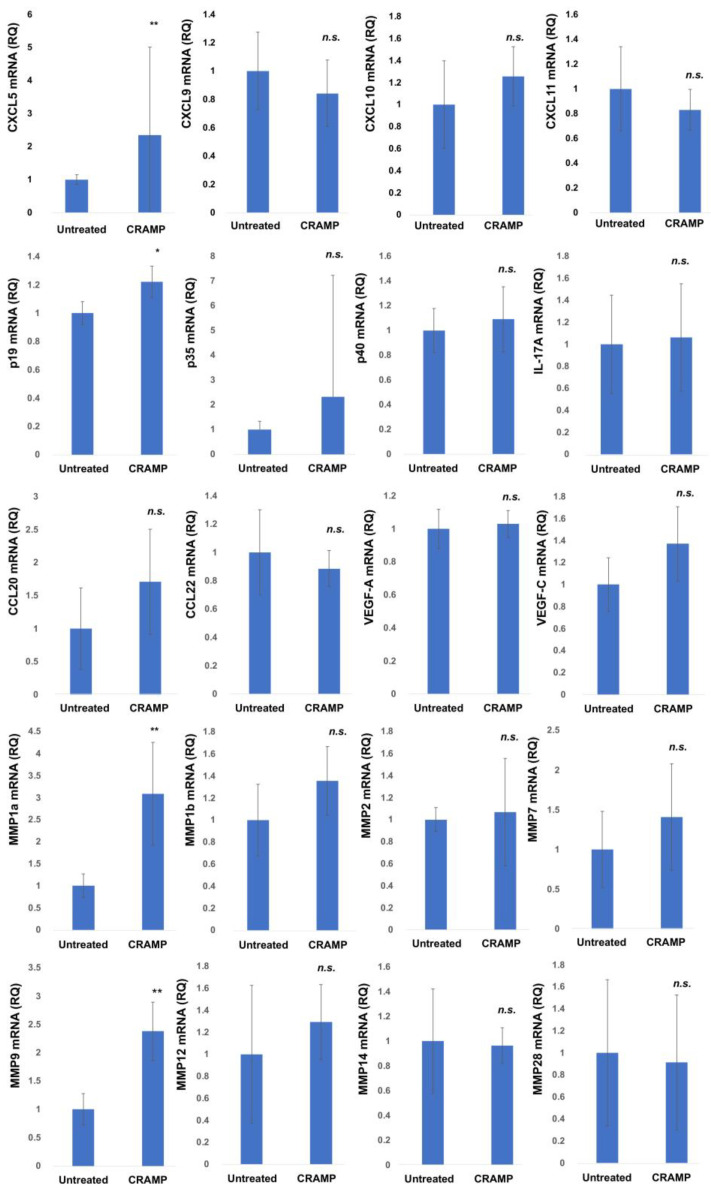
Expression of chemokines, cytokines, and MMPs mRNA in B16F10 melanoma was analyzed by quantitative RT-PCR using the ΔΔCt method (n = 15). The data from each donor were obtained by triplicate assays, and then the mean ± SD was calculated (c). * marks a significant (*p* < 0.05) difference. ** marks a significant (*p* < 0.01) difference. n.s.: not significant.

To investigate the immunomodulatory effects of LL-37 on the tumor microenvironment in vivo, the mouse B16F10 melanoma model was used. The peritumoral administration of LL-37 significantly increased the expression of *CXCL5*, *IL23A*, *MMP1a*, and *MMP9* mRNA in the tumor microenvironment (Figure 2). There were no significant differences in the expression of *CCL20*, *CCL22*, *CXCL9*, *CXCL10*, *CXCL11*, *VEGF-A*, *VEGF-C*, *IL-17A*, *IL-12p35*, *IL-12p40*, *MMP1b*, *MMP2*, *MMP7*, *MMP12*, *MMP14*, and *MMP28*. The averages of the data from 15 mice are shown in Figure 2.

LL-37 increased the mRNA expression and protein production of CXCL5, IL-23p19, and MMP-9 in A375 human melanoma cell lines. n.s.: not significant

**Figure 3:**
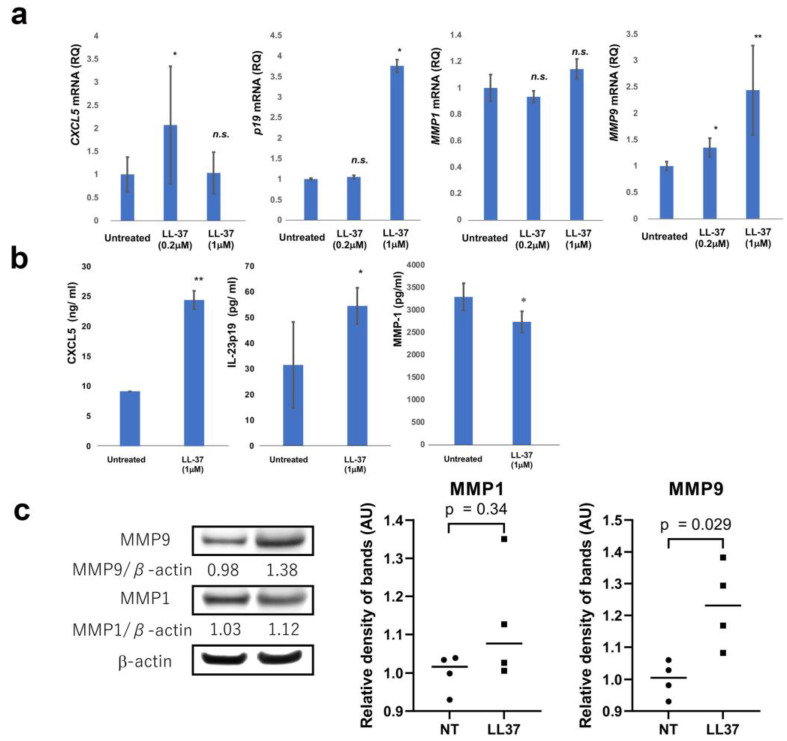
Expression of *IL23A*, *CXCL5*, *MMP1*, and *MMP9* mRNA in A375 melanoma cells stimulated with or without LL-37 were analyzed by quantitative RT-PCR using the ΔΔCt method (n = 3). The data from each donor were obtained by triplicate assays, and then the mean ± SD was calculated. (**a**) Culture supernatant from A375 was harvested as described in Materials and Methods and measured by ELISA (n = 3) (**b**). MMP-1 and MMP-9 production was analyzed by Western blotting (**c**). Data from each donor were obtained from triplicate assays, and mean ± SD values were calculated. Representative data from at least three independent experiments are shown. * marks a significant (*p* < 0.05) difference. ** marks a significant (*p* < 0.01) difference. The uncropped blots are shown in Figure S1. n.s.: not significant.

Next, to evaluate the LL-37-reactive cells in the melanoma microenvironment, the direct immunomodulatory effects of LL-37 on melanoma cells were evaluated. A375 melanoma cells were stimulated with LL-37, and the mRNA expression of *CXCL5*, *IL23A*, *MMP1*, and *MMP9* were evaluated in vitro. The mRNA expression of *CXCL5*, *IL23A*, and *MMP9* on A375 melanoma cells were significantly increased by LL-37, whereas there was no significant difference of MMP-1 with LL-37 stimulation (Figure 3a). To validate the increased mRNA expression of *CXCL5*, *IL23A*, and *MMP9* on A375, the protein production of CXCL5, *IL23A*, and MMP-9 by A375 cells was investigated using an ELISA assay and Western blot. The production of CXCL5 and IL-23p19 by A375 cells was significantly increased by LL-37 stimulation (Figure 3b). The production of MMP-9 by A375 was significantly increased by LL-37 (Figure 3c). There was no significant increase in MMP-1 production by A375 (Figure 3b,c).

LL-37 increased the mRNA expression and protein production of CXCL5, IL-23p19, and MMP-1 in monocyte-derived M2 macrophages in vitro.

**Figure 4:**
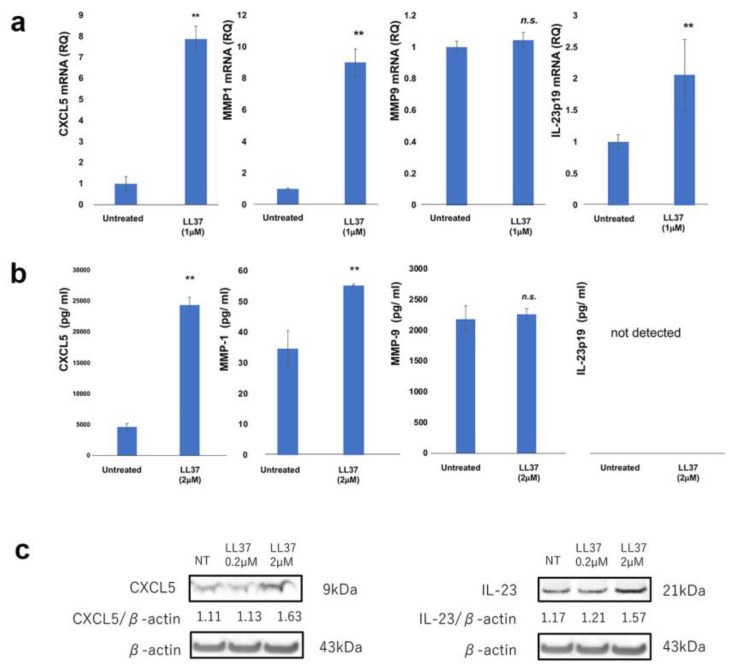
Expression of *IL23A*, *CXCL5*, *MMP1*, and *MMP9* mRNA in M2 macrophages stimulated with or without LL-37 were analyzed by quantitative RT-PCR using the ΔΔCt method (n = 3). The data from each donor were obtained by triplicate assays, and then the mean ± SD was calculated. (**a**) Culture supernatant from M2 macrophages was harvested as described in Materials and Methods and measured by ELISA (n = 3). Data from each donor were obtained from triplicate assays, and mean ± SD values were calculated. Representative data from at least three independent experiments are shown (**b**). ** marks a significant (*p* < 0.01) difference. CXCL5 and IL-23p19 production was analyzed by Western blotting (**c**). The uncropped blots are shown in Figure S2. n.s.: not significant.

Since LL-37 was located at CD163+ M2 macrophages (Figure 1c), we further hypothesized that LL-37 increases the mRNA expression and production of angiogenetic factors (CXCL5, IL-23p19, MMP1, MMP9) from human CD163+ M2 macrophages. To test this hypothesis, monocyte-derived M2 macrophages were stimulated by LL-37 in vitro. LL-37 increased *CXCL5*, *MMP1* and *IL23A* mRNA expression, but it did not increase MMP9 mRNA in vitro (Figure 4a). To validate the increased mRNA expression of *CXCL5*, *MMP1*, and *IL23A* on M2 macrophages, the protein production of CXCL5, MMP-1, and IL-23p19, as well as MMP-9, from M2 macrophages was investigated using an ELISA assay. Similar to mRNA expression, the production of CXCL5, MMP-1 (Figure 4b), and IL-23p19 (Figure 4c) was significantly increased by LL-37 stimulation, whereas there was no significant difference in MMP-9 production by M2 macrophages (Figure 4b).

LL-37 increased the angiogenetic activity of A375.

**Figure 5:**
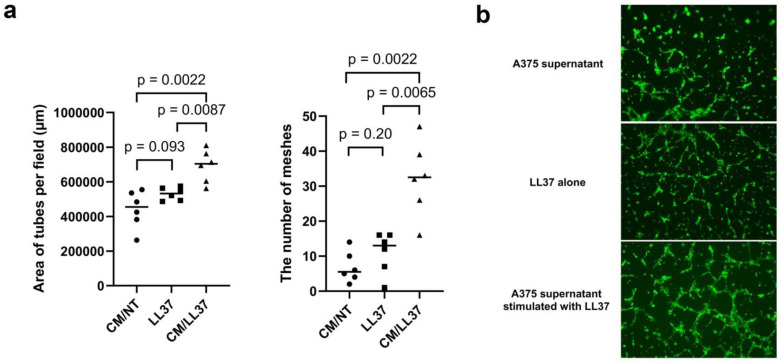
The tube formation assay was performed by applying HDMECs treated with culture supernatant of recombinant LL-37-treated A375 or culture medium with or without recombinant LL-37 onto the Matrigel and incubating for 24 h. To eliminate the effect of proliferation, cells were treated with mitomycin C before the assay (**a**). Representative images are shown (**b**) (n = 5 for each group).

Since LL-37 increased *CXCL5* and *IL23A* mRNA expression, as well as CXCL5 and IL-23p19 production, and since LL-37 increased only MMP-1 production from M2 macrophages, the pro-angiogenic activities of A375-related factors were further examined. To address this issue, an in vitro angiogenesis assay with Matrigel was used. HDMECs treated with LL-37-stimulated A375 culture supernatant formed tube networks, whereas HDMECs treated with LL-37 showed no tube networks under the same culture condition (Figure 5a). To objectively evaluate the activity of angiogenesis, the mesh area was calculated, and tube formation was counted using a BZ-X800 microscope. Tube formation area per field was significantly higher in HDMECs treated with A375+LL-37 culture supernatant than in HDMECs treated with or without LL-37 (Figure 5b).

## 4. Discussion

LL-37 is expressed in various skin cancers [14,15], and it even promotes the immunosuppressive microenvironment through the production of immunosuppressive chemokines by tumor-associated macrophages (TAMs) [15]. Since T stage determines the overall survival (OS) of melanoma patients [26], and since the expression of LL-37 was increased in parallel with T stage of melanoma as seen in the present study, it is important to understand the tumor-promoting effects of LL-37 on melanoma and its stromal cells. Indeed, peritumoral injection of CRAMP (the mouse ortologe of LL-37) increased the mRNA expression of *CXCL5*, *IL23A*, *MMP1*, and *MMP9* in B16F10 melanoma in vivo, suggesting that LL-37 could enhance pro-angiogenesis in melanoma. Moreover, mRNA expression and protein production of these pro-angiogenetic factors was increased in A375 melanoma cells as well as CD163+ M2 macrophages by the stimulation of LL-37 in vitro. Notably, tube formation assay revealed that LL-37 increased angiogenetic activity of A375. Correctively, LL-37-induced CXCL5, IL-23p19, MMP-1 and MMP-9 could promote angiogenetic activity in melanoma, leading to the local invasion of melanoma which contribute to T stage in melanoma patients.

Among the pro-angiogenetic factors described above, IL-23p19 plays one of the central roles in promoting tumor growth in various cancers through its pro-angiogenesis activity [9]. Indeed, IL-23p19 increased the angiogenetic activity of cutaneous angiosarcoma [8]. In addition, IL-23p19 directly or indirectly promotes the infiltration of myeloid cells such as M2 macrophages and neutrophils, leading to the increased secretion of TGF-β, IL-10, and VEGF, and thus promoting angiogenesis in breast cancer [27]. Moreover, IL-23p19 increases IL-23p19 receptor expression on macrophages and enhances macrophage-mediated angiogenesis in hepatocellular carcinoma [28]. Notably, in our present study, IL-23p19 was increased in both melanoma cells and CD163+ M2 macrophages. Since recombinant IL-23p19 forms favorable tube networks in vitro [8], IL-23p19 could, at least in part, promote angiogenetic activity in tumor microenvironment in melanoma.

CXCL5 is a chemokine that promotes tumor formation by triggering the migration of CXCR2+ immune cells to tumors [29,30]. Not only recruiting immunosuppressive cells, such as precursors of TAMs, neutrophils, and myeloid-derived suppressor cells in the tumor microenvironment [30], CXCL5 can recruit endothelial cells (ECs) via its highly conserved glutamic acid-leucine-arginine ‘ELR’ motif [29,31], promoting cancer progression in various cancer types [20]. For example, the interaction between ECs and cancer cells enhances EC recruitment and promotes cancer progression through the EGFR-NF-kB-CXCL5-CXCR2 pathway in bladder cancer [32]. In addition, CXCL5 is positively correlated with the micro-vessel marker CD31, and it activates the AKT/NF-kB/FOXD1/VEGF-A pathway to enhance its tube formation ability in a CXCR2-dependent manner in colorectal cancer [33]. Moreover, CXCL5-overexpressing melanoma cells recruited high amounts of neutrophils and exhibited significantly increased lymphangiogenesis in a mouse melanoma xenograft model [34]. Notably, IL-17A promotes CXCR2-dependent angiogenesis in liver cancer [35]. Since IL-23p19 plays important roles in inducing Th17 cell proliferation even in the cancer microenvironment [36,37], increased levels of CXCL5 in parallel with IL-23p19 by LL-37 could trigger angiogenesis, leading to local invasion of melanoma in the primary lesion.

MMP-9, as well as MMP-1, is a pro-angiogenesis factor, and they could both be prognostic biomarkers for melanoma [38]. Indeed, high expression of MMP-9 protein, as well as infiltration of TAMs, was detected in proximity to intravascular pillars [33]. Notably, MMP-9 inhibition blocked formation of pillars in vessels, and the inhibition of MMP-9 promotes abrogated pillar formation in melanoma [39], leading to suppression of angiogenesis in melanomas. TAMs also produce various MMPs, which play critical roles in the tissue remodeling associated with protein cleavage to modify the immune microenvironment, angiogenesis, tissue repair, local invasion, and metastasis [40]. Notably, MMPs are among the central angiogenetic factors associated with M2-polarized TAMs in skin cancers [40]. For example, osteopontin signaling increased the secretion of MMP-9 from TAMs to promote angiogenesis and tumor progression in a melanoma model [41]. Interestingly, in our present study, LL-37 increased the protein production of MMP-9 from A375 melanoma cells, whereas LL-37 increased MMP-1 from M2 macrophages in vitro, suggesting that LL-37 might promote tumor invasion not only by the stimulation of melanoma cells but also by the stimulation of TAMs in melanoma microenvironment. In aggregate, our present study also suggested the pro-angiogenetic roles of TAMs through IL-23p19, CXCL5 and MMP-1 in melanoma.

## 5. Conclusions

**Figure 6:**
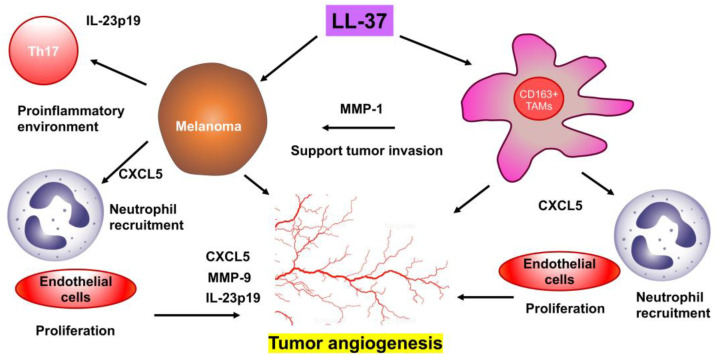
Summary of the present study.

Correctively, LL-37-induced CXCL5, IL-23p19, MMP-1 and MMP-9 could promote angiogenetic activity in melanoma tumor microenvironment, leading to the local invasion of melanoma which contributes to T stage in melanoma patients. Therefore, targeting LL-37, MMP-9, and TAMs could be a potential anti-angiogenic drug target to suppress the local invasion of melanoma cells, which might improve the OS of melanoma patients in the future (Figure 6).

## Figures and Tables

**Table 1:** Characteristics of patients with melanoma.

		Age	Sex	Subtype	Tumor Thickness (mm)	TNM	Stage
	case 1	69	M	acral	0	TisN0M0	0
	case 2	78	F	acral	0	TisN0M0	0
	case 3	72	F	acral	0	TisN0M0	0
	case 4	67	M	acral	0	TisN0M0	0
	case 5	75	F	acral	0	TisN0M0	0
Tis	case 6	49	F	acral	0	TisN0M0	0
	case 7	86	F	acral	0	TisN0M0	0
	case 8	69	F	acral	0	TisN0M0	0
	case 9	85	M	acral	0	TisN0M0	0
	case 10	21	F	non-CSD	0	TisN0M0	0
	case 11	78	F	CSD	0.5	T1aN0M0	IA
	case 12	79	F	CSD	0.5	T1aN0M0	IA
	case 13	62	M	acral	0.35	T1aN0M0	IA
	case 14	65	M	acral	0.90	T1aN0M0	IA
	case 15	48	F	acral	0.90	T1bN0M0	IB
T1	case 16	36	F	acral	0.60	T1aN0M0	IA
	case 17	43	F	acral	0.80	T1aN0M0	IA
	case 18	72	M	acral	0.42	T1aN0M0	IA
	case 19	75	M	acral	0.70	T1bN0M0	IB
	case 20	65	M	CSD	0.05	T1bN0M0	IA
	case 21	64	F	acral	1.02	T2bN0M0	IIA
	case 22	46	F	acral	1.05	T2aN0M0	IB
	case 23	81	F	CSD	1.20	T2aN0M0	IB
	case 24	88	F	CSD	1.50	T2bN0M0	IIA
	case 25	72	M	acral	1.80	T2bN0M0	IIA
T2	case 26	65	M	non-CSD	1.50	T2aN0M0	IB
	case 27	42	F	acral	1.80	T2aN0M0	IB
	case 28	69	F	non-CSD	1.03	T2aN0M0	IB
	case 29	21	F	acral	1.30	T2aN0M0	IB
	case 30	21	F	non-CSD	1.3	T2aN0M0	IB
	case 31	47	M	acral	3.5	T3aN3cM0	IIIC
	case 32	63	F	acral	3	T3aN0M0	IIA
	case 33	83	F	CSD	2	T3aN0M0	IIA
	case 34	62	F	CSD	3	T3aN1aM0	IIIB
	case 35	79	M	acral	3.5	T3aN1bM0	IIIB
T3	case 36	79	F	acral	3.2	T3aN0M0	IIA
	case 37	64	F	acral	2	T3bN1aM0	IIIC
	case 38	84	F	acral	2	T3aN0M0	IIA
	case 39	81	F	acral	3	T3aN0M0	IIA
	case 40	71	M	non-CSD	3	T3aN0M0	IIA
	case 41	74	F	acral	6	T4bN0M0	IIC
	case 42	82	F	acral	6	T4aN0M0	IIB
	case 43	77	M	acral	6	T4aN0M0	IIB
	case 44	90	F	CSD	4.4	T4aN0M0	IIB
	case 45	69	F	acral	4.5	T4bN1aM0	IIIC
T4	case 46	39	M	CSD	9	T4bN2aM0	IIIC
	case 47	70	M	acral	14	T4bN0M0	IIC
	case 48	35	M	non-CSD	20	T4bN1aM1b	IV
	case 49	84	M	acral	6	T4bN0M0	IIC
	case 50	47	M	CSD	8	T4bN2bM0	IIIC

(CSD: chronic sun damage).

## Data Availability

All data generated or analyzed during this study are included in this article. Further enquiries can be directed to the corresponding author.

## Supplementary Materials

The following supporting information can be downloaded at: https://www.mdpi.com/article/10.3390/cancers15061678/s1, Figure S1: Raw data for Figure 3; Figure S2: Raw data for Figure 4.
