# Supplementary material for: LL-37 Might Promote Local Invasion of Melanoma by Activating Melanoma Cells and Tumor-Associated Macrophages
Source: Cancers (Basel). 2023 Mar 9;15(6):1678. doi: 10.3390/cancers15061678 (PMC10046113; doi:10.3390/cancers15061678)
Supplement: Supplementary file 1 [file cancers-15-01678-s001.zip › cancers-2086122-supplementary.pdf]

Supplementary Material: LL-37 Might Promote Local Invasion of Melanoma by Activating Melanoma Cells and Tumor-Associated Macrophages

Kentaro Ohuchi, Tetsuya Ikawa, Ryo Amagai, Toshiya Takahashi, Yuna Roh, Junko Endo, Yumi Kambayashi, Yoshihide Asano and Taku Fujimura

MMP9

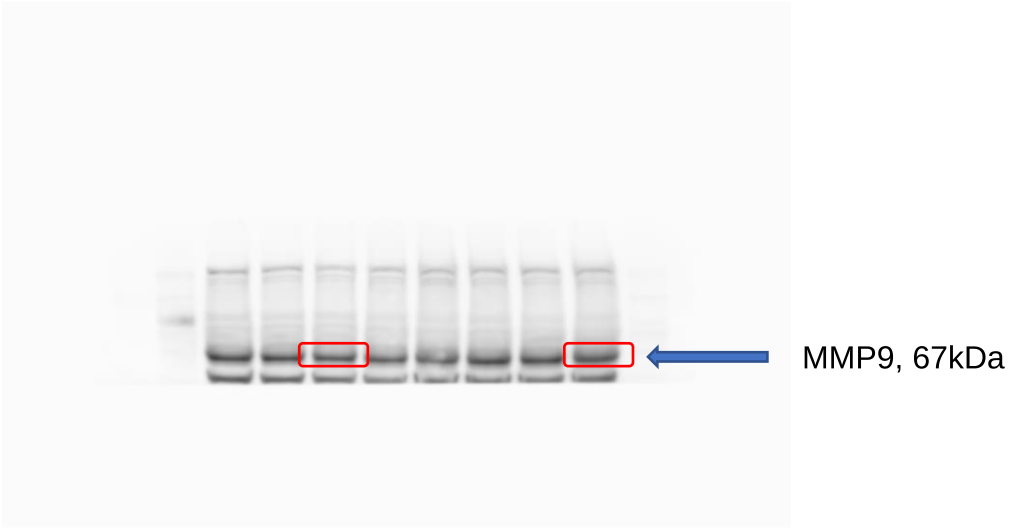

MMP1

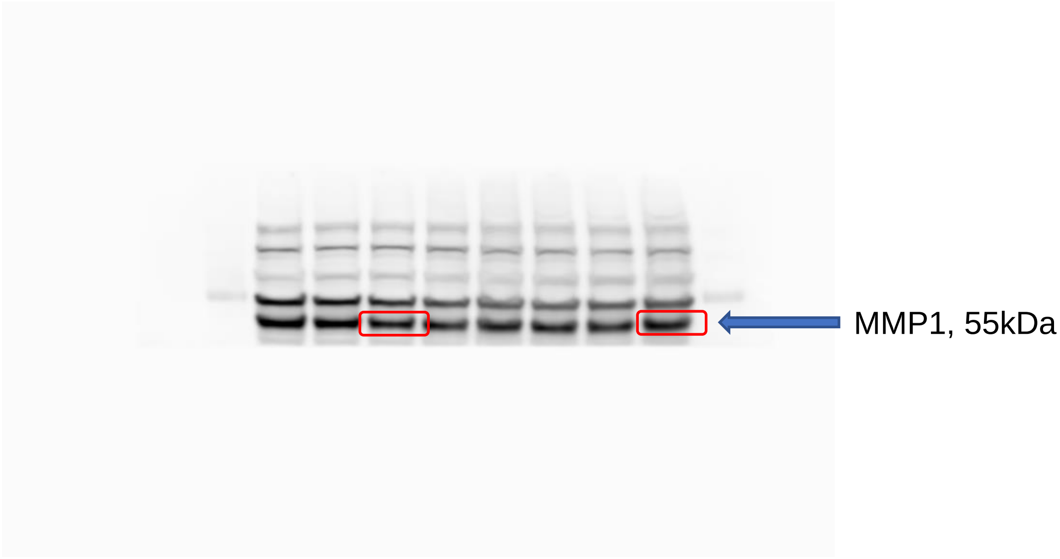

Figure S1. Raw data for Figure 3

CXCL5

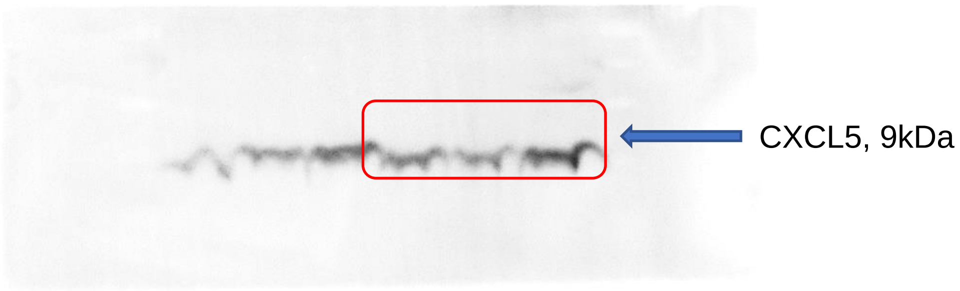

IL-23

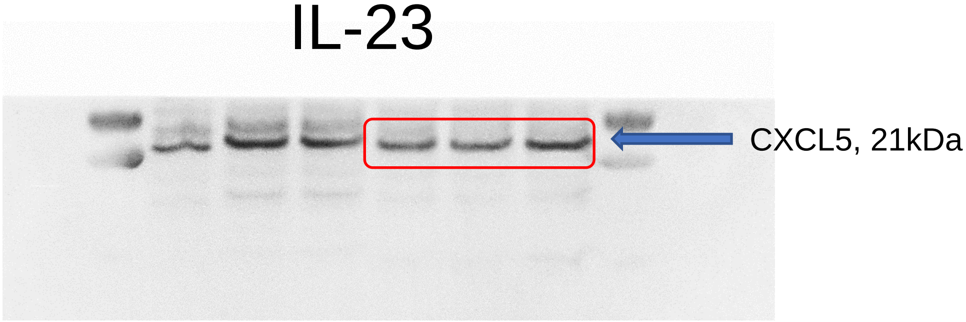

Figure S2. Raw data for Figure 4
